# Application of miR-193a/WT1/PODXL axis to estimate risk and prognosis of idiopathic membranous nephropathy

**DOI:** 10.1080/0886022X.2019.1642210

**Published:** 2019-07-29

**Authors:** Wei Zhang, Yeping Ren, Jie Li

**Affiliations:** Department of Nephrology, The Second Affiliated Hospital of Harbin Medical University, Harbin, China

**Keywords:** miR-193a, Wilms tumor type 1, podocalyxin, idiopathic membranous nephropathy, renal survival, receiver operating characteristic curve

## Abstract

**Background:** This investigation was managed to explore whether miR-193a in combination with two podocytes, namely, Wilms tumor type 1 (WT1) and podocalyxin (PODXL), were feasible in estimating onset and prognosis of idiopathic membranous nephropathy (IMN).

**Methods:** We recruited a total of 189 healthy controls and 364 IMN patients, whose urine samples were prepared to measure the expression of miR-193a and PODXL. Meanwhile, renal tissues collected from above-mentioned IMN patients (*n* = 364) and renal cell carcinoma patients (*n* = 189) were arranged to determine the expression of WT1. Ultimately, receiver operating characteristic curves were constructed to appraise the performance of miR-193a, WT1, and PODXL in predicting renal survival of IMN patients.

**Results:** The IMN patients were measured with up-regulated miR-193a expression and down-regulated WT1/PODXL expression, when compared with healthy controls (*p* < 0.05). Moreover, highly expressed miR-193a, lowly expressed WT1/PODXL, elevated amounts of proteinuria (>3.79 g/24 h)/serum creatinine (>174.63 μmol/L), and declined GFR (≤68.13 mL/min/1.73 m^2^) were implicated as prominent biomarkers for the poor renal survival of IMN patients (all *p* < 0.05). Notably, miR-193a combined with PODXL and WT1 generated optimal effects in differentiating IMN patients from healthy controls (AUC = 0.994) and also in anticipating the renal survival state of IMN patients (AUC = 0.824), when compared with strategies that merely employed ≤2 of the biomarkers.

**Conclusion:** The combination of miR-193a, WT1, and PODXL might serve as a favorable strategy for expecting IMN prognosis.

## Introduction

Membranous nephropathy (MN), a principal account for nephrotic syndrome among global adults, was documented with an annual rate of around 1.2/10^5^ [1], and ∼2/3 of the MN cases were defined as idiopathic membranous nephropathy (IMN) for their murky pathogenesis [2]. It was statistically informed that merely 30%–60% of the IMN patients were spontaneously relieved [3,4], whereas others who failed to respond to immune depressive therapies tended to progress into end-stage renal failure (ESRF) [5,6]. The mental stress and economic burden brought by ESFR were enormous, which necessitated exploration of eligible biomarkers for IMN treatment [7,8]. Of note, IMN was explicitly proposed as a disease featured by antigen-specificity of podocyte membrane [9], and injury of podocytes could give rise to proteinuria, a characteristic manifestation of IMN [10]. These linkages of podocytes with IMN suggested that etiologies underlying malfunctioning of podocytes might also explain the onset of IMN.

MiRNAs, a category of small non-coding RNAs composed of 22–25 nucleotides, were highly engaged in modulating pathophysiologic mechanisms by suppressing expression of target genes, which was realized through blockage of protein translation or induction of mRNA degradation [11]. Notably, expressional variations of miRNAs have been broadly documented during onset and progression of podocyte-related nephrotic disorders [12–14]. For instance, the urinary levels of miR-146a and miR-155 were raised within systemic lupus nephritis patients [12], and the amounts of plasma miR-125b and miR-186 were lessened with the alleviation of focal segmental glomerulosclerosis (FSGS) [14]. Additionally, intentionally elevating miR-193a expression was found to promote extensive disappearance of foot processes and thereby FSGS onset within mice models [15]. Given the facts that IMN and FSGS shared certain pathological traits, such as injured cytoskeletons and slit diaphragms of podocytes, it was conjectured that biomarkers (e.g., miR-193a) that played a core role in FSGS development could also matter in the pathogenesis of IMN. Intriguingly, the facilitating effects of miR-193a on nephropathy were, to some extent, dependent on suppression of Wilm’s tumor protein (WT1) [15], which was usually suggestive of quantitatively reduced podocytes and damaged glomerular filtration barrier [16,17]. The WT1 also functioned as a regulator for podocalyxin (PODXL) [18], which was capable of preventing adhesion of foot processes and maintaining regular structure of podocytes [19]. Summing up the above, miR-193a, WT1, and PODXL were, respectively, accountable for disordered functioning of podocytes, suggesting themselves as promising biomarkers for proteinuria-related nephropathies (e.g., IMN) [20].

Despite the seemingly plausible deductions as mentioned above, few researches have been performed to associate miR-193a, WT1, and PODXL with onset and prognosis of IMN patients. To fill in this blank, we aimed to explore the value of miR-193a, WT1, and PODXL in diagnosing IMN and predicting IMN prognosis among a Chinese population, which might offer solid evidences for clinical treatment of IMN in future.

## Materials and methods

### Inclusion of IMN patients

From March 2014 to May 2018, 364 patients that were histopathologically confirmed with IMN were recruited from Renal Bio-resources Collaborative Study System in China (China READY). The IMN patients were further classified into stage I (*n* = 162), stage II (*n* = 130), and stages III + IV (*n* = 72), according to the guidelines formulated in 2001 for pathological diagnosis of renal biopsy [21]. The included IMN cases all conformed to the following criteria: (1) they were clinically diagnosed with proteinuria, hematuria, hypertension, and edema; (2) their light microscopy results included slightly stiff glomerular capillary loop and vacuolar-like modification of basement membrane; (3) their immunofluorescent results were specified as granular deposition along the capillary wall, which was mainly dominated by IgG and C3; and (4) their electron microscope results indicated that electronic dense deposits were visible on the epithelial side of early stage basement membrane, and foot processes were extensively fused. Meanwhile, 189 healthy controls who took medical examinations were also gathered, and they showed normal levels of blood routine, urine routine, liver function, renal function, blood glucose, and blood lipids. Furthermore, the participants were excluded if: (1) they were diagnosed with tumor-associated MN, hepatitis B virus-associated nephritis, lupus nephritis or drug-associated nephropathy; (2) they developed complications, such as diabetes mellitus; (3) they showed infection-relevant symptoms in the past month; (4) their alanine aminotransferase/aspartate aminotransferase level was twice higher than the normal value; and (5) they were females in the pregnant/lactation period. All the included participants or their direct relatives have signed informed consents, and this investigation has obtained approval from the second Affiliated Hospital of Harbin Medical University, the institutional review board of the second Affiliated Hospital of Harbin Medical University and the ethics committee of the second Affiliated Hospital of Harbin Medical University and Renal Bio-resources Collaborative Study System in China (China READY).

### Calculation of glomerular filtration rate (GFR)

The simplified modification of diet in renal disease formula as follows [22] was employed to estimate GFR (mL/min/1.73 m^2^) of individuals: *a* × 186 × serum creatinine (Scr, mg/dL)^−1.154^ × age (years old)^−0.203^ (*a* = 1 for males and *a* = 0.742 for females).

### Treatment and follow-up of IMN patients

The treatment strategies for IMN patients were designated according to their proteinuria level. In particular, the IMN patients whose proteinuria level ranged between 3.5 and 6 g/d were given angiotensin converting enzyme inhibitor (ACEI), angiotensin II receptor antagonist (ARB), and low-protein diet. Besides, those carrying a proteinuria level of ≥6 g/d were given prednisone based on the standard of 0.6 mg/(kg × d), and ones who weighed >70 kg were administrated with 40 mg/d prednisone. Ultimately, the hospitalized IMN patients were given combined treatments of heparin sodium, ACEI, ARB, and liver care.

In addition, the IMN patients were followed up from the date when renal biopsy was performed until December 1, 2018 or the date when subjects died or their renal function deteriorated. Their renal function was considered to deteriorate when (1) Scr level became > 445 μmol/L, or (2) Scr level detected at the endpoint of follow-up was more than twice of the value detected at the beginning.

### Reverse transcription-polymerase chain reaction (RT-PCR) for determination of miR-193a

The clean midstream urine gathered from enrollees was centrifugated at 3000 g for 30 min, so that cells and cell debris could be removed. Then the total RNAs within exosomes were extracted complying with Trizol method (Takara, Japan), and their purity and concentration were evaluated with the assistance of UV spectrophotometer. Subsequently, the total RNAs were reversely transcribed into cDNAs following the guidance of TaqMan microRNA reverse transcription kit (Applied Biosystems, USA). Aided by primers (Ambion, USA) of miR-193a (sense: 5′-TGGGTCTTTGCGGGCGAGATGA-3′, anti-sense: 5′-ACCCAGAAACGCCCGCTCTACT-3′), and U6 (sense: 5′-GCTTCGGCAGCACATATACTAAAAT-3′, anti-sense: 5′-CGCTTCACGAATTTGCGTGTCAT-3′), PCR was performed under conditions of (1) 50 °C for 2 min, (2) 95 °C for 10 min, and (3) 50 cycles of 95 °C for 15 s and 60 °C for 1 min, in the light of procedures enlisted in the TaqMan microRNA assay kit (Applied Biosystems, USA). Eventually, expressions of target genes were analyzed on the ABI Prism 7000 SDS software (Applied Biosystems, USA), and they were quantified based on the 2^–ΔΔCt^ method.

### Western blotting for measurement of WT1

The renal tissues were gathered from the recruited IMN patients who underwent percutaneous renal biopsy, and the normal renal tissues were obtained from renal cell carcinoma patients (*n* = 189) who received nephrectomy. Then total protein was extracted from tissues through addition of lysis buffer, and its concentration was determined in line with Braford method. Subsequently, total protein (50 μg) was prepared for performing sodium dodecyl sulfate-polyacrylamide gel electrophoresis (SDS-PAGE) on the electrophoresis system (Hoefer Mighty Small, USA), and semi-dry electrophoresis transfer device (Bio-Rad, USA) was managed to transfer proteins from gel to nitrocellulose membrane (Pall, USA). After 2-h blockage of the membrane at room temperature, the rabbit anti-human primary antibodies (Abcam, USA) against WT1 (1:1000, Cat. No.: ab89901), and β-actin (1:1000, Cat. No.: ab8227) were supplemented for overnight incubation at 4 °C. With Tris Buffered Saline Tween employed to rinse the mixture for three times, the goat anti-rabbit secondary antibodies labeled by horse radish peroxidase (HRP) (1:5000, Cat. No.: ab97080, Abcam, USA) were added to implement another 2-h incubation at room temperature. Besides, enhanced chemi-luminescence kit (Thermo Fisher Scientific, USA) was adopted to evaluate the development results, and images were collected via a photo scanner (model: 1650, Epson, Japan). Finally, the gray scale of bands was semi-quantified based on BandLeader 3.0 software (Magnitec, Israel).

### Detection of PODXL with enzyme-linked immunosorbent assay (ELISA)

The mid-portion urine was taken from each participant in the morning, and the urine residues were obtained after centrifugation at 2000 r/min for 10 min. With utilization of ELISA kit (Shanghai Yueyan Biotechnology, China), the PODXL level within urine residues was quantified based on absorbance (A) values that were measured at the wavelength of 450 nm on the enzyme labeling apparatus (model: Varioskan Flash, Thermo Scientific, USA).

### Statistical analyses

The statistical analyses throughout this manuscript were accomplished with utilization of SPSS 19.0 software. On one hand, the continuous variables (mean ± standard deviation) that conformed to normal distribution were compared based on student’s t test or one-way analysis (ANOVA). On the other hand, the categorical variables in the form of number (*n*) were compared through implementation of chi-square test. Furthermore, Spearman correlation analysis was conducted to assess the correlations between miR-193a/WT1/PODXL expressions and clinical traits of IMN patients. The Kaplan-Meier survival curves were also established to describe the renal survival of IMN patients, with log-rank test adopted for between-group comparisons. Moreover, parameters that influenced IMN prognosis were estimated by performing univariate/multivariate cox-regression analyses, and the receiver operating characteristic (ROC) curves were constructed to assess the performance of miR-193a, WT1, and PODXL in diagnosing IMN and in predicting IMN prognosis. It was deemed as statistical significance in case of *p* < .05.

## Results

### Comparison of clinical features between IMN patients and healthy controls

The age and sex ratios were quite matched among healthy controls, IMN patients at stage I, IMN patients at stage II, and IMN patients at stage III + IV (all *p* > .05) (Table 1). Furthermore, the IMN patients were detected with higher levels of proteinuria, blood urea nitrogen (BUN), total cholesterol, triglyceride, and Scr than healthy controls (*p* < .05), By contrast, the GFR and serum albumin level became lessened among IMN patients, when compared with healthy controls (*p* < .05). More than that, IMN patients at the advanced stages (i.e., III + IV) revealed higher proteinuria, total cholesterol, triglyceride, and Scr levels, as well as lower GFR and serum albumin level, than those classified into stages I and II (all *p* < .05).

**Table 1.** Comparison of baseline characteristics between IMN patients and healthy controls.

Clinical characteristics	Healthy control	IMN patients		
		Stage I	Stage II	Stage III + IV
Number (*n*)	189	162	130	72
Age (years old)	47.28 ± 9.74	46.85 ± 10.68	49.04 ± 11.05	49.75 ± 11.05
Gender (*n*)				
Female	86	64	57	30
Male	103	98	73	42
Proteinuria (g/24 h)	–	2.89 ± 0.67	4.01 ± 1.31#	5.42 ± 1.75#&
Serum Albumin (g/L)	48.24 ± 5.31	36.13 ± 6.01*	27.33 ± 5.08*#	19.76 ± 5.04*#&
BUN (mmol/L)	4.68 ± 1.39	6.17 ± 2.11*	6.48 ± 1.93*	6.93 ± 1.75*
GFR (mL/min/1.73 m^2^)	131.46 ± 12.71	80.65 ± 13.12*	65.44 ± 10.97*#	44.81 ± 11.26*#&
Total cholesterol (mmol/L)	4.16 ± 1.13	6.27 ± 2.84*	10.85 ± 3.09*#	13.28 ± 5.22*#&
Tryglyeride(mmol/L)	1.08 ± 0.36	1.59 ± 0.61*	1.94 ± 0.56*#	2.38 ± 0.55*#&
Scr (μmol/L)	75.96 ± 13.45	93.42 ± 16.82*	162.83 ± 25.14*#	378.68 ± 57.91*#&

BUN: blood urea nitrogen; GFR: glomerular filtration rate; Scr: serum creatinine.

*Compared with Healthy Control Group, the *p* values <.05.

#Compared with Stage I, the *p* values <.05.

&Compared with Stage II, the *p* values <.05.

### *Correlation of miR-193a*, *WT1*, *and PODXL expressions with clinical features of IMN patients*

**Figure 1.**
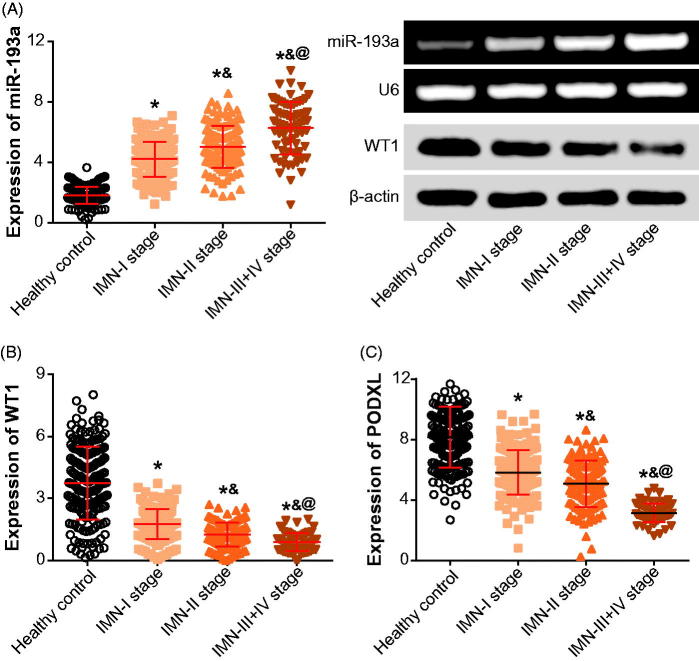
The miR-193a (A), WT1 (B), or PODXL (C) expressions were compared between IMN patients and healthy controls. PODXL: podocalyxin; IMN: idiopathic membranous nephropathy. *p < .05 when compared with healthy controls; &*p* < .05 when compared with IMN patients at stage I; @*p* < .05 when compared with IMN patients at stage II.

Up-regulated expression of miR-193a and down-regulated expression of PODXL were observed within urine samples of IMN patients, as compared with those obtained from healthy controls (*p* < .05) (Figure 1). Moreover, renal tissues gathered form IMN patients exhibited lower WT1 expressions than normal tissues that were excised form renal cell carcinoma patients (*p* < .05). Moreover, IMN patients of stage III + IV exhibited higher miR-193a expression than those of stage I and II (*p* < .05), yet WT1 and PODXL expressions were decreased more significantly within IMN patients at advanced stages than within those of stage I and II (*p* < .05).

**Figure 2.**
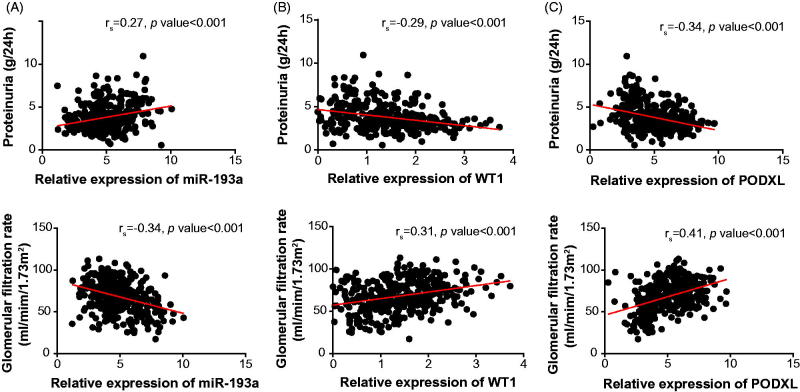
The correlation between miR-193a (A), WT1 (B), or PODXL (C) expressions and proteinuria level and GFR among IMN patients. PODXL: podocalyxin; IMN: idiopathic membranous nephropathy.

Additionally, with the mean expression of miR-193a (i.e., 4.91) as the cutoff level, the IMN patients were divided into highly expressed (>4.91) miR-193a group (*n* = 219) and lowly expressed (≤4.91) miR-193a group (*n* = 145). Abiding by the same principle, the identical IMN cohort was additionally separated into highly expressed (>1.41) WT1 group (*n* = 139) and lowly expressed (≤1.41) WT1 group (*n* = 225), as well as highly expressed (>5.03) PODXL group (*n* = 154) and lowly expressed (≤5.03) PODXL group (*n* = 210). It was demonstrated that the IMN patients that carried up-regulated miR-193a expression and down-regulated WT1/PODXL expressions were associated with higher odds of incremental proteinuria level (>3.79 g/24 h), increased Scr amount (>174.63 μmol/L) and declined GFR (≤68.13 mL/min/1.73 m^2^) than those carrying down-regulated miR-193a expression and up-regulated WT1/PODXL expressions (Table 2). Besides, expressions of miR-193a (Figure 2(A)), WT1 (Figure 2(B)) and PODXL (Figure 2(C)) all presented significant correlations with proteinuria level (miR-193a: *r*_s_ = 0.27, WT1: *r*_s_ = –0.29, PODXL: *r*_s_ = –0.34), and GFR (miR-193a: *r*_s_ = –0.34, WT1: *r*_s_ = 0.31, PODXL: *r*_s_ = 0.41) among the investigated IMN patients.

**Table 2.** Association of miR-193a, WT1, and PODXL expressions with the clinical indicators of IMN patients.

Clinical characteristics (*N* = 364)	miR-193a expression*				WT1 expression@				PODXL expression#			
	High (>4.91)	Low (≤4.91)	*χ*^2^	*p* value	High (>1.41)	Low (≤1.41)	*χ*^2^	*p* value	High (>5.03)	Low (≤5.03)	*χ*^2^	*p* value
Age (years old, *n*)												
>48.21	137	93			84	146			103	127		
≤48.21	82	52	0.09	.760	55	79	0.73	.392	51	83	1.57	.211
Gender (*n*)												
Female	93	58			61	90			55	96		
Male	126	87	0.22	.640	78	135	0.53	.465	99	114	3.66	.056
Proteinuria (g/24 h, *n*)												
>3.79	143	77			74	146			83	137		
≤3.79	76	68	5.43	.020	65	79	4.88	.027	71	73	4.78	.029
Serum Albumin (g/L, *n*)												
>29.75	113	87			66	134			91	109		
≤29.75	106	58	2.49	.115	73	91	5.06	.025	63	101	1.85	.173
BUN (mmol/L, *n*)												
>6.43	107	82			68	121			88	101		
≤6.43	112	63	2.07	.150	71	104	0.81	.368	66	109	2.91	.088
GFR (mL/min/1.73 m^2^, *n*)												
>68.13	124	101			99	126			108	117		
≤68.13	95	44	6.28	.012	40	99	8.44	.004	46	93	7.82	.005
Total cholesterol (mmol/L, *n*)												
>9.29	118	89			83	124			95	112		
≤9.29	101	56	2	.157	56	101	0.74	.389	59	98	2.53	.112
Tryglyeride (mmol/L, *n*)												
>1.87	121	94			88	127			96	119		
≤1.87	98	51	3.31	.069	51	98	1.68	.196	58	91	1.18	.277
Scr (μmol/L, *n*)												
>174.63	108	56			52	112			59	105		
≤174.63	111	89	4.03	.045	87	113	5.31	.021	95	105	4.90	.027

IMN: Idiopathic membranous nephropathy; BUN: blood urea nitrogen; GFR: glomerular filtration rate; Scr: serum creatinine.

*The whole IMN cohort (*n* = 364) were divided into highly expressed miR-193a group (>mean miR-193a expression) and lowly expressed miR-193a group (≤mean miR-193a expression).

@The collected 364 IMN patients were categorized into highly expressed WT1 group (>mean WT1 expression) and lowly expressed WT1 group (≤mean WT1 expression).

#The identical IMN cohort (*n* = 364) were grouped into ones with highly expressed PODXL (>mean PODXL expression) and ones with lowly expressed PODXL (≤mean PODXL expression).

### *Association of miR-193a*, *WT1*, *and PODXL expressions with renal survival of IMN patients*

**Figure 3.**
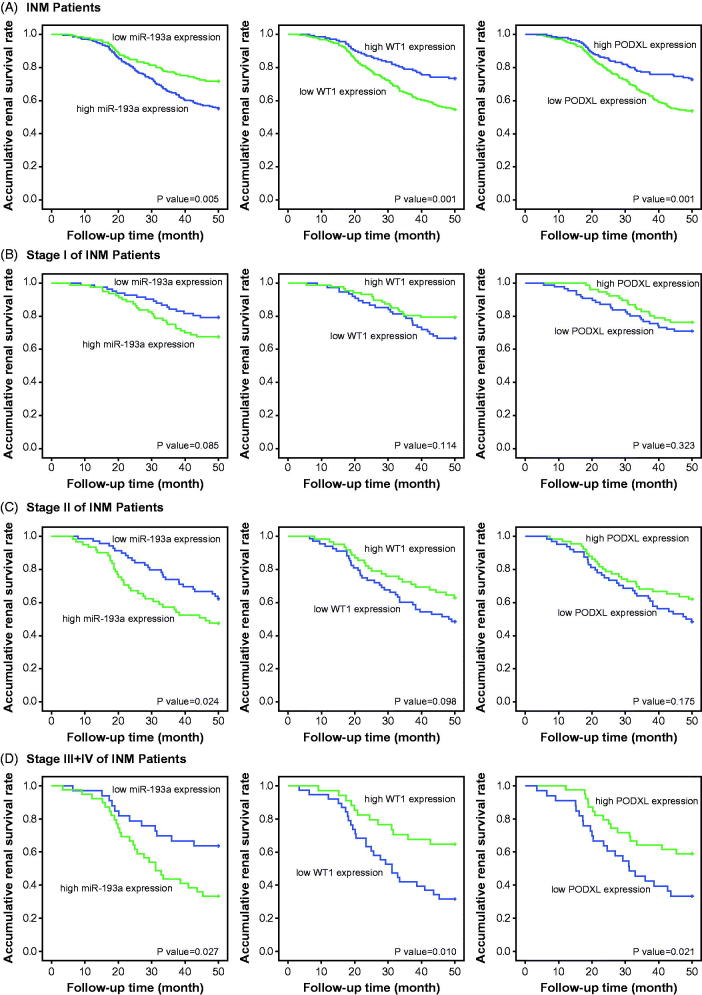
Regression analysis of miR-193a, WT1, or PODXL levels for renal survival among IMN patients (A), IMN patients at stage I (B), IMN patients at stage II (C), and IMN patients at stages III + IV (D). PODXL: podocalyxin; IMN: idiopathic membranous nephropathy.

With the whole IMN population as the study subjects, over-expressed miR-193a, along with under-expressed WT1 and PODXL, could be deemed as significant predictors for the unfavorable renal survival of IMN patients, when compared with under-expressed miR-193a and over-expressed WT1/PODXL (all *p* < .05) (Figure 3(A)). Nevertheless, the expressional change of miR-193a, WT1, or PODXL failed to discriminate the renal survival of IMN patients at stage I (*p* > .05) (Figure 3(B)), whereas up-regulated miR-193a expression appeared to reflect the ideal renal survival of IMN population at stage II (*p* < .05) (Figure 3(C)). Interestingly, among the IMN population of stage III + IV, miR-193a, WT1, and PODXL were differentially expressed between ones with favorable outcome and those with poor renal survival (*p* < .05) (Figure 3(D)). The cox regression analyses (Table 3) also implied highly expressed miR-193a, lowly expressed WT1/PODXL, high-level proteinuria, large amounts of Scr, and low GFR as vital indicators of IMN patients’ unfavorable renal survival.

**Table 3.** Association of miR-193a, WT1, and PODXL expressions with renal survival of IMN patients.

Items	Univariate analysis			Multivariate analysis		
	Hazard ratio	95% CI	*p* value	Hazard ratio	95% CI	*p* value
miR-193a expression*						
High (>4.91) vs. Low (≤4.91)	2.05	1.31–3.22	.002	1.91	1.17–3.11	.010
WT1 expression@						
High (>1.41) vs. Low (≤1.41)	0.44	0.28–0.69	<.001	0.49	0.30–0.81	.005
PODXL expression#						
High (>5.03) vs. Low (≤5.03)	0.44	0.28–0.68	<.001	0.47	0.28–0.76	.002
Age (years old)						
>48.21 vs. ≤48.21	1.37	0.88–2.13	.168	1.46	0.90–2.37	.129
Gender						
Female vs. Male	0.97	0.63–1.49	.885	0.88	0.55–1.42	.605
Proteinuria (g/24 h)						
>3.79 vs. ≤3.79	2.24	1.43–3.53	<.001	1.77	1.09–2.88	.022
Serum Albumin (g/L)						
>29.75 vs. ≤29.75	0.78	0.51–1.19	.244	0.78	0.49–1.26	.312
BUN (mmol/L)						
>6.43 vs. ≤6.43	0.90	0.59–1.38	.639	1.00	0.62–1.60	.987
GFR (mL/min/1.73 m^2^)						
>68.13 vs. ≤68.13	0.40	0.24–0.61	<.001	0.55	0.34–0.88	.014
Total cholesterol (mmol/L)						
>9.29 vs. ≤9.29	1.05	0.68–1.60	.836	1.23	0.77–1.97	.395
Tryglyeride (mmol/L)						
>1.87 vs. ≤1.87	0.90	0.59–1.39	.645	1.06	0.66–1.70	.819
Scr (μmol/L)						
>174.63 vs. ≤174.63	2.07	1.35–3.18	.001	1.51	0.94–2.42	.087

IMN: Idiopathic membranous nephropathy; BUN: blood urea nitrogen; GFR: glomerular filtration rate; CI: confidence interval; Scr: serum creatinine.

*The whole IMN cohort (*n* = 364) were divided into highly expressed miR-193a group (>mean miR-193a expression) and lowly expressed miR-193a group (≤mean miR-193a expression).

@The collected 364 IMN patients were categorized into highly expressed WT1 group (>mean WT1 expression) and lowly expressed WT1 group (≤mean WT1 expression).

#The identical IMN cohort (*n* = 364) were grouped into ones with highly expressed PODXL (>mean PODXL expression) and ones with lowly expressed PODXL (≤mean PODXL expression).

### Application of ROC curves to evaluate roles of miR-193a, WT1 and PODXL in predicting renal survival of IMN patients

**Figure 4.**
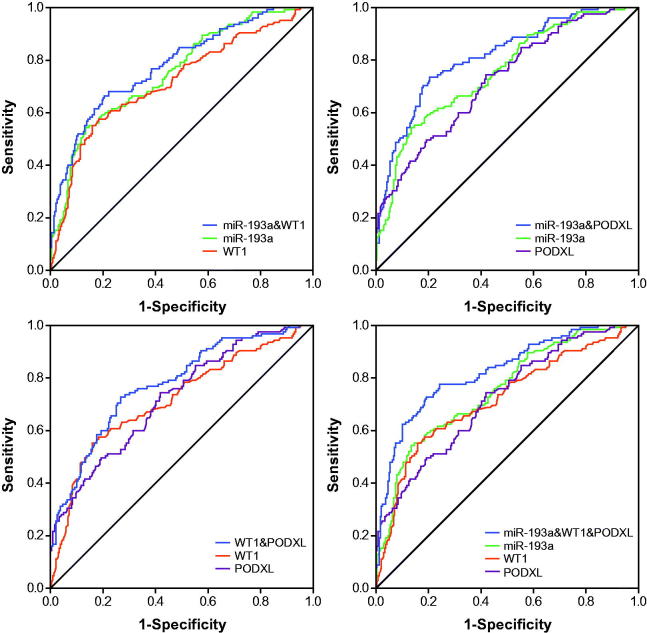
Respective and combined contributions of miR-193a, WT1, and PODXL to evaluating renal survival of the whole IMN patients.

**Figure 5.**
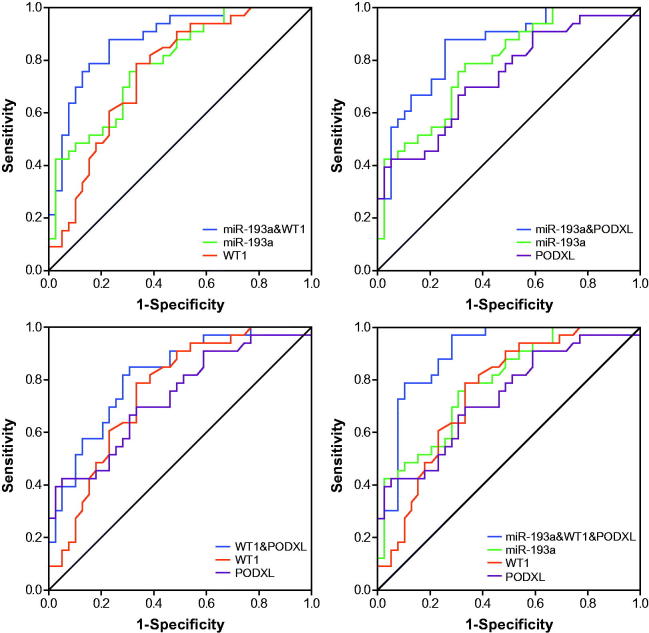
Respective and combined roles of miR-193a, WT1, and PODXL in assessing renal survival of IMN patients at stage III + IV.

**Figure 6.**
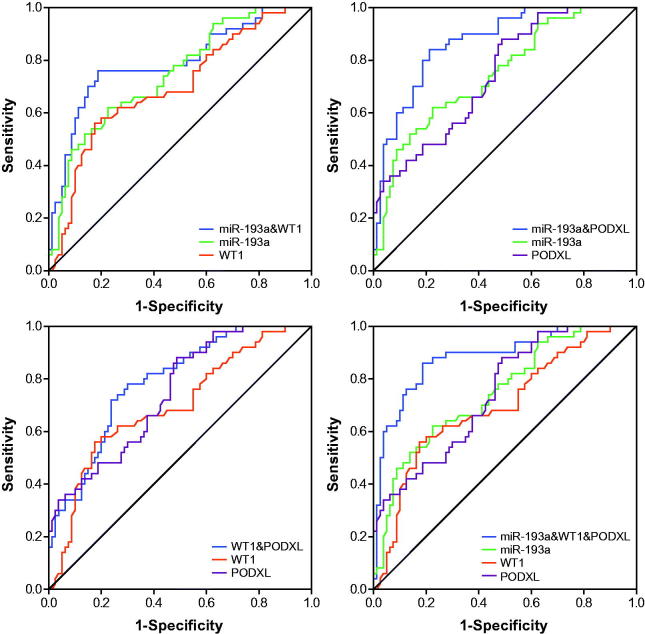
Respective and combined contributions of miR-193a, WT1, and PODXL to appraising renal survival of IMN patients at stage II.

**Figure 7.**
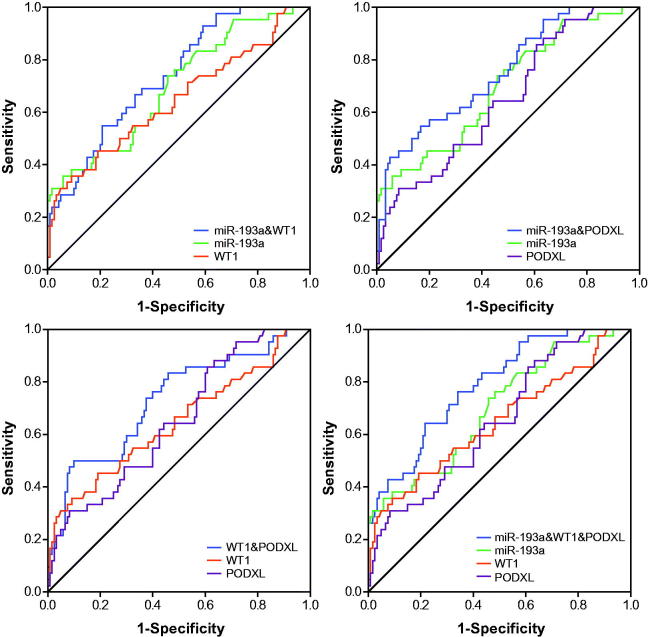
Respective and combined capability of miR-193a, WT1, and PODXL in evaluating stage-I IMN patients’ renal survival.

**Figure 8.**
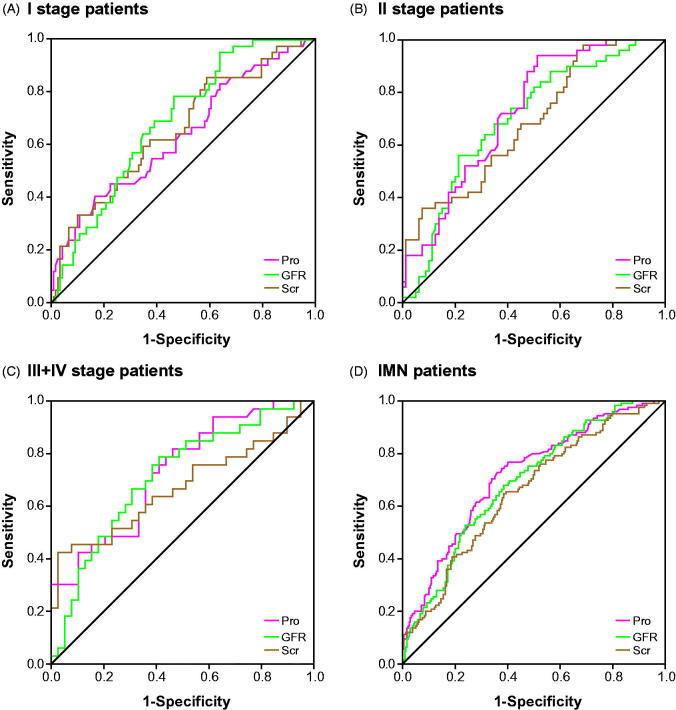
Respective contributions of proteinuria (Pro), GFR, and Scr to evaluating renal survival of IMN patients at stage I (A), IMN patients at stage II (B), and IMN patients at stages III + IV (C) and the whole IMN patients (D).

The miR-193a possessed an optimal accuracy (AUC = 0.753) in estimating the prognosis of the whole IMN population, and PODXL (AUC = 0.723) and proteinuria (AUC = 0.718) were, respectively, ranked as second and third (Table 4 and Figures 4 and 8). Besides, expressional alterations of miR-193a (AUC = 0.788), which was followed by WT1 (AUC = 0.754) and PODXL (AUC = 0.734), topped in appraising the renal survival of IMN patients at advanced stages (i.e., III + IV) (Table 4 and Figures 5 and 8). Moreover, GFR (AUC = 0.997) seemed as a preferred choice in evaluating the renal survival of IMN patients at stage II (Table 4 and Figures 6 and 8), whereas miR-193a, WT1, PODXL, proteinuria, GFR, and Scr, possessed similar capabilities in assessing the renal survival of IMN patients at stage I (Table 4 and Figures 7 and 8). Furthermore, miR-193a combined with PODXL (AUC = 0.813) stood out in forecasting the renal survival of all IMN patients, when compared with miR-193a combined with WT1 (AUC = 0.781) and WT1 combined with PODXL (AUC = 0.776) (Table 5 and Figure 4). Notably, miR-193a in combination with WT1 and PODXL (AUC = 0.824) exhibited the highest AUC value in expecting IMN prognosis, irrespective of the IMN population studied (Table 5 and Figures 4–7).

**Table 4.** Respective values of miR-193a, WT1, PODXL, proteinuria, GFR, and Scr for appraising renal survival of IMN patients.

Biomarkers	IMN patients	Value	Sensitivity	Specificity	AUC	95%CI
miR-193a	Stage I	1.05	0.357	0.908	0.696	0.603–0.790
	Stage II	5.29	0.620	0.775	0.747	0.661–0.832
	Stage III + IV	6.22	0.788	0.667	0.788	0.686–0.891
	All*	5.77	0.544	0.866	0.753	0.701–0.805
WT1	Stage I	5.54	0.357	0.942	0.646	0.542–0.750
	Stage II	1.00	0.560	0.825	0.696	0.603–0.790
	Stage III + IV	0.91	0.788	0.667	0.754	0.643–0.866
	All*	1.00	0.552	0.841	0.717	0.660–0.775
PODXL	Stage I	3.46	0.857	0.392	0.650	0.557–0.743
	Stage II	4.67	0.880	0.512	0.743	0.660–0.827
	Stage III + IV	7.07	0.424	0.949	0.734	0.618–0.850
	All*	4.81	0.744	0.582	0.723	0.669–0.777
Proteinuria	Stage I	3.36	0.405	0.833	0.634	0.534–0.734
	Stage II	3.45	0.940	0.487	0.725	0.640–0.811
	Stage III + IV	4.85	0.818	0.538	0.732	0.617–0.846
	All*	3.50	0.728	0.640	0.718	0.663–0.773
GFR	Stage I	82.85	0.786	0.533	0.687	0.602–0.772
	Stage II	60.90	0.560	0.787	0.997	0.606–0.789
	Stage III + IV	48.52	0.788	0.590	0.714	0.594–0.834
	All*	67.70	0.680	0.615	0.690	0.635–0.745
Scr	Stage I	85.65	0.857	0.408	0.657	0.560–0.754
	Stage II	142.26	0.960	0.337	0.684	0.592–0.776
	Stage III + IV	419.47	0.424	0.974	0.671	0.540–0.801
	All*	142.26	0.648	0.615	0.656	0.598–0.714

IMN: Idiopathic membranous nephropathy; GFR: glomerular filtration rate; Scr: serum creatinine; AUC: area under the curve; CI: confidence interval

*With the whole IMN patients considered, including those at stages I, II, and III + IV.

**Table 5.** The Combined action of miR-193a, WT1, and PODXL for evaluating renal survival of IMN patients.

Combination biomarkers	IMN patients	Sensitivity	Specificity	AUC	95%CI
miR-193a&WT1	Stage I	0.548	0.792	0.739	0.656–0.821
	Stage II	0.760	0.812	0.791	0.707–0.874
	Stage III + IV	0.879	0.769	0.880	0.802–0.959
	All*	0.664	0.795	0.781	0.731–0.831
miR-193a&PODXL	Stage I	0.548	0.833	0.752	0.668–0.837
	Stage II	0.840	0.787	0.869	0.809–0.929
	Stage III + IV	0.879	0.744	0.853	0.767–0.940
	All*	0.736	0.795	0.813	0.766–0.859
WT1&PODXL	Stage I	0.500	0.900	0.730	0.638–0.821
	Stage II	0.720	0.762	0.788	0.710–0.865
	Stage III + IV	0.848	0.692	0.817	0.721–0.914
	All*	0.728	0.732	0.776	0.725–0.826
miR-193a&WT1&PODXL	Stage I	0.643	0.783	0.786	0.709–0.862
	Stage II	0.860	0.812	0.882	0.820–0.943
	Stage III + IV	0.970	0.718	0.898	0.824–0.972
	All*	0.728	0.808	0.824	0.779–0.870

IMN: Idiopathic membranous nephropathy; AUC: area under the curve; CI: confidence interval.

*With the whole IMN patients considered, including those at stages I, II, and III + IV.

### The diagnostic efficacy of miR-193a, WT1, and PODXL for IMN patients

The miR-193a (AUC = 0.976) appeared to be more productive than WT1 (AUC = 0.876) and PODXL (AUC = 0.826) in differentiating IMN patients from healthy controls (Supplementary Table 1), and the variation of proteinuria content could effectively separate IMN patients at stage I from those at stage II (AUC = 0.786) or stage III + IV (AUC = 0.922). Besides, miR-193a combined with WT1 generated a satisfactory accuracy in diagnosing IMN patients at stage III + IV from those at stage I (AUC = 0.929) or stage II (AUC = 0.776), when compared with other combination groups made up of two molecules (Supplementary Figures 1–3 and Supplementary Table 2). Interestingly, miR-193a combined with PODXL and WT1 led to higher accuracies in differentiating IMN patients of various stages (I vs. II: AUC = 0.799, I vs. III + IV: AUC = 0.957, II vs. III + IV: AUC = 0.807, IMN vs. control: 0.994) than any other diagnostic patterns (Supplementary Figure 4 and Supplementary Table 2).

## Discussion

Exosomes were principally referred to double-layer microvesicles that were fused by multivesicular endosomes and cytomembranes, and they were broadly available within human body fluid, including blood, urine, and cerebrospinal fluid [23,24]. An accumulating body of evidence has confirmed the involvement of exosomes in renal pathophysiology, including glomerular disorders (e.g., FSGS, IgA nephropathy, and DN), tubular diseases (e.g., Bartter syndrome and Gitelman syndrome), acute kidney injury, renal fibrosis, and polycystic kidney. Notably, certain biomarkers (e.g., miRNAs) were selectively enriched and degraded during shaping of urinary exosomes [23–25], which implied that expressional changes of pivotal biomarkers were capable of mirroring the metergasis of kidney. Moreover, the double-membrane structure of exosomes could guarantee high-level integrity and stability of miRNAs within exosomes owing to their resisting RNase-induced degradation [23,26], which stressed the high sensitivity of miRNAs in reflecting changes of the microenvironment. Considering the traits of urinary biomarkers as mentioned above, disease-specific miRNAs could serve as promising candidates for judging onset and progression of nephropathies (e.g., IMN).

Within this investigation, urinary miR-193a was insinuated as a biomarker for IMN development, since that miR-193a expression within urine was obviously promoted with the increase of IMN severity (*p* < .05) (Figure 1). Besides, there presented a remarkable correlation between miR-193a expression and proteinuria level among the studied IMN patients (Figure 2). This correlation might further emphasize the reliability of miR-193a in reflecting IMN deterioration, allowing for that the variation range of proteinuria level was a fine reflector of IMN histopathology [27]. Nonetheless, apart from FSGC [14] and IMN, plenty of podocyte injury-related disorders failed to trigger expressional change of miR-193 within golmerulus, including HIV-associated nephropathy, diabetic nephropathy, puromycin nephropathy, adriamycin nephropathy, suPAR model, Heyman nephritis, protamine sulfate injection, and Alport syndrome [15]. These discrepancies might, from another aspect, illustrate the outstanding specificity of miR-193 in estimating onset and prognosis of IMN (Table 4), yet relevant accounts for the underlying mechanisms were still ambiguous. Additionally, the high-degree stability of exosome-sourced miR-193 could also explain the superior sensitivity of miR-193 in indicating IMN occurrence and development (Table 4). Virtually, biomarkers for IMN (e.g., miR-193) were highly worthy of exploration, for that glomerular diseases, whose treatment demanded adoption of chronic renal replacement therapy [28], have become an essential constituent among renal disorders within the past 20 years [29].

What’s more, two podocytes located downstream of miR-193 (i.e., WT1 and PODXL) were also nephropathy-relevant [30]. For one thing, mutation of WT1 could induce idiopathic diffuse mesangial sclerosis and Denys-Drash syndrome [31], and depression of WT1 expression tended to trigger onset of crescentic nephritis and mesangial sclerosis [16]. For another, patients with FSGS, minimal lesions, diabetic nephropathy, and IgA nephropathy were discovered with a reduction in the glomerular expression of PODXL [32]. Besides the renal disorders, our investigation further proposed down-regulated expressions of WT1 and PODXL as desirable indicators for incremental risk (Supplementary Figures 1–4 and Supplementary Tables 1–2) and unfavorable prognosis (i.e., renal survival) of IMN (Figure 3 and Table 3). As a matter of fact, the roles of WT1 and PODXL in altering the structure of podocytes could, to some degree, explain the clinical association of WT1/PODXL with IMN onset [33]. To be specific, WT1-interacting protein (i.e., WTIP) possibly led to dysregulated phenotypes of podocytes by hindering expressions of WT1-dependent genes [34]. Furthermore, the proper functioning of PODXL not merely ensured the integrity of negative-charge barrier in the glomerular filtration membrane, but also could maintain the normal slit diaphragm between foot processes [35]. From the above, WT1 and PODXL could participate in certain IMN-related mechanisms that miR-193a was not involved with, which might account for why miRNA-193a combined with WT1 and PODXL produced higher accuracies in assessing IMN onset and prognosis than miRNA-193a alone (Figure 4 and Table 5).

Conclusively, miR-193a in combination with WT1 and PODXL might serve as a desirable manner in appraising IMN onset and prognosis, and this combined diagnosis was also able to tell apart the prognosis of IMN patients at various stages. Despite the above-mentioned strengths, the current investigation failed to figure out how miR-193a/WT1/PODXL axis functioned underlying the etiology of IMN. Moreover, the results of this study might not fit for IMN patients of non-Chinese ethnicities, since that the prognosis of IMN patients could differ among different ethnicities [36,37]. Besides, application of distinct treatment strategies might contribute to bias in terms of evaluating IMN prognosis, so it might be more reasonable to recruit IMN patients who received uniform treatments. Finally, the results of this study could be more clinically valuable, if the expression of WT1 within urinary exosomes, rather than renal tissues, was examined [38]. Above all, all these aspects as mentioned above should be settled in future.
